# Advances in MXene Materials: Fabrication, Properties, and Applications

**DOI:** 10.3390/ma18214894

**Published:** 2025-10-25

**Authors:** Subin Antony Jose, Jordan Price, Jessica Lopez, Erick Perez-Perez, Pradeep L. Menezes

**Affiliations:** Department of Mechanical Engineering, University of Nevada-Reno, Reno, NV 89557, USA; subinj@unr.edu (S.A.J.); jdprice@unr.edu (J.P.); jessicalopez@unr.edu (J.L.); erickp@unr.edu (E.P.-P.)

**Keywords:** MXenes, etching and exfoliation, surface chemistry, surface termination, surface functionalization, hydrophilicity

## Abstract

This review provides a critical overview of MXenes, an innovative class of 2D transition metal carbides, nitrides, and carbonitrides, emphasizing their synthesis, properties, and application potential. We systematically examine synthesis methods, contrasting top-down approaches with emerging green alternatives and bottom-up techniques, evaluating each in terms of scalability, cost, and environmental impact. This paper highlights MXenes’ unique characteristics, including high electrical conductivity, tunable surface chemistry, and structural versatility, which enable their use in energy storage, environmental remediation, biomedicine, and electromagnetic shielding. Key challenges such as oxidative instability, interfacial incompatibility, and hazardous etching processes are critically discussed. We identify future research priorities, including defect-engineered stabilization, AI-optimized manufacturing, and advanced integration protocols to bridge the gap between laboratory breakthroughs and industrial deployment. By integrating these insights, this review offers a roadmap for advancing MXenes from laboratory innovation to industrial application.

## 1. Introduction

MXenes represent a novel class of 2D materials with the general chemical formula M_n+1_X_n_T_x_, where M is the early transition metal (Ti, V, Nb, Mo, etc.) that forms the structural backbone, X is the carbon and/or nitrogen atoms that create strong covalent bonds with M, n is the integer (1–4) representing the number of X layers between M layers, and T_x_; is the surface termination groups (–O, –OH, –F) introduced during synthesis. These materials are derived from MAX phases (M_n+1_AX_n_), where A is group 13–16 elements (Al, Si, etc.) that are selectively etched out. The same M, X, and n variables apply as in MXenes [1,2,3]. The transformation from MAX to MXene occurs through controlled etching processes that remove the A layers while preserving the M-X framework. This creates atomically thin sheets with (a) layered morphology: stacked 2D crystals with adjustable interlayer spacing (0.3–1.2 nm), (b) functionalized surfaces: termination groups that dictate chemical behavior, and (c) high surface area: theoretical values up to 300 m^2^/g for single layers [4,5].

The etching process (originally using HF acid, now including safer alternatives) creates surfaces that naturally terminate with –O, –OH, or –F groups, which influence electronic properties (–O terminations enhance conductivity), chemical stability (–F groups improve oxidation resistance), and hydrophilicity (–OH groups enable water dispersibility) [6].

Since the first synthesis in 2011 (Ti_3_C_2_T_x_ from Ti_3_AlC_2_), the MXene family has grown to include over 50 compositions with varying M and X combinations. Theoretical studies suggest hundreds more are possible by combining different transition metals (M), adjusting carbon/nitrogen ratios (X), and engineering termination group mixtures (T_x_) [4]. This structural versatility enables precise property tuning for applications ranging from energy storage to biomedical devices, making MXenes one of the most adaptable material families in nanotechnology [7].

What distinguishes MXenes from other 2D materials is their unique combination of metallic conductivity (up to 15,000 S/cm), inherent hydrophilicity (contact angle < 10°), and mechanical robustness (Young’s modulus ~0.5 TPa) [2,8]. These properties stem from their electronic structure, where transition metal d-orbitals enable charge transport, and their tunable surface chemistry, which facilitates covalent functionalization impossible in graphene [7]. Such characteristics have led MXenes into diverse applications ranging from ultrahigh-capacity batteries (exceeding 1000 mAh/g) to biomedical sensors with single-molecule detection capabilities [9,10].

The evolution of MXene research has progressed through three key phases: (1) initial exploration of fundamental properties (2011–2015), (2) development of application-specific modifications (2015–2020), and (3) current focus on solving scalability and stability challenges for commercialization. This review systematically examines these aspects, beginning with a critical analysis of synthesis protocols, contrasting top-down etching (e.g., HF, molten salts) with emerging bottom-up techniques like chemical vapor deposition (CVD). We then explore the structure–property relationships that enable applications in energy storage, electromagnetic interference (EMI) shielding (effectiveness > 60 dB at 1μm thickness), and photothermal therapy. Despite remarkable progress, persistent challenges in oxidative stability (<7 days in ambient air) and industrial-scale production (current cost > $500/g) necessitate innovative solutions. Recent advances in atomic layer deposition coatings and continuous flow reactors show promise for overcoming these limitations.

This review article brings together insights drawn mainly from existing review articles and key findings from selected recent primary studies. By doing so, our goal is to provide a clear and comprehensive overview of advances in MXene, as well as emerging directions that appear across the literature. Unlike many previous reviews that focus solely on either synthesis or applications, this work adopts an integrated perspective. We connect synthesis, stability, and scalability, showing how processing choices impact long-term performance, environmental footprint, safety, and cost. We also highlight practical solutions, including protective coatings, self-passivating formulations, and continuous-flow reactors, providing a roadmap for translating MXene research from the laboratory to industrial-scale deployment.

This review concludes with a forward-looking perspective on MXene commercialization, emphasizing the need for cross-disciplinary collaboration to realize their potential in addressing global energy and environmental challenges.

## 2. Synthesis and Manufacturing of MXenes

### 2.1. Top-Down and Bottom-Up Synthesis of MXenes

MXene synthesis strategies are broadly categorized into top-down and bottom-up approaches, each offering distinct advantages for different applications [11]. The top-down approach involves selectively etching the A element from MAX phases, while the bottom-up approach constructs MXenes atom by atom without relying on MAX phases.

Top-down synthesis is the most widely used method for MXene production due to its simplicity and reliability. This process involves chemically etching the ‘A’ element from MAX phase precursors (typically Ti_3_AlC_2_, Mo_2_TiAlC_2_, etc.) using HF acid or alternative etchants like fluoride salts (NH_4_HF_2_) or molten salts (ZnCl_2_), resulting in layered transition metal carbides or nitrides. In typical laboratory procedures, 1 g of Ti_3_AlC_2_ MAX phase is immersed in 20–50 mL of 10–50 wt.% HF at 25–40 °C for 24–48 h under continuous stirring (300–500 rpm), followed by multiple washing and centrifugation cycles (3500 rpm for 5–10 min per cycle) until it reaches neutral pH (6–7) [12]. This three-stage process begins with high-purity MAX phase synthesis (1350–1600 °C), followed by selective A-layer removal (48 h in 10–50% HF), and concludes with delamination via intercalation and sonication (30–60 min at 100 W) to yield single-layer flakes [13]. Typical yields of delaminated MXene from MAX precursors range between 35–50 wt.%, depending on etchant concentration and washing efficiency [14]. Recent advances in continuous flow reactors have scaled production to 100 g batches while maintaining flake uniformity (D90 < 2 μm) and controlled surface terminations (–O/–OH ratio).

In contrast, bottom-up techniques like chemical vapor deposition (CVD) and molecular beam epitaxy (MBE) enable atomic-level precision for specialized applications but remain limited by low yields (<100 mg/batch) and high equipment costs (>$1 M). These methods excel in creating heterostructures (MXene/graphene) and defect-free monolayers for electronic applications but face significant scalability challenges [14]. Despite its potential, bottom-up synthesis faces significant challenges, including higher costs and difficulties in scaling up production. As a result, it is primarily limited to laboratory-scale research or the development of novel MXene variants [15]. Table 1 below summarizes key differences among top-down, bottom-up, and advanced synthesis methods of MXene.

Emerging hybrid approaches combine both paradigms, using top-down flakes as seeds for bottom-up lateral growth, to achieve both scalability and atomic precision. Recent hybrid protocols have employed plasma-enhanced CVD (PECVD) at 600–700 °C with 20–50 sccm precursor flow for 30–45 min to deposit uniform layers on pre-etched MXene flakes, achieving sub-nanometer thickness control [18]. Researchers focus on plasma-enhanced CVD for roll-to-roll production and machine-learning-optimized etching parameters to bridge the gap between laboratory research and industrial deployment. The choice between methods ultimately depends on application requirements: top-down for bulk applications like conductive coatings or energy storage, and bottom-up for precision applications in nanoelectronics or quantum devices. These emerging advanced MXene synthesis methods, including bottom-up, hybrid, and green approaches, offer unique advantages and challenges compared to conventional top-down etching. Bottom-up techniques such as CVD and MBE provide atomic-level control over stoichiometry, defect density, and heterostructure formation, making them ideal for electronic, optoelectronic, and quantum device applications. However, these methods are limited by low yields (<100 mg/batch), extremely high equipment costs, and scalability challenges, restricting their use primarily to laboratory-scale research [4]. Hybrid approaches provide a balance between scalability and atomic precision. These methods enable control over flake size and surface terminations, making them suitable for energy storage, flexible electrodes, and catalytic applications. However, multi-step complexity and limited reproducibility remain key concerns [19]. Environmentally friendly or “green” methods, including electrochemical etching in mild electrolytes, molten salt etching, and hydrothermal alkaline etching, reduce or eliminate the use of hazardous HF and offer safer and more sustainable alternatives. While these approaches produce MXenes with unique surface chemistries (e.g., Cl^−^ or OH– terminated) suitable for biomedical, environmental, or aqueous applications, they face challenges in controlling yield, flake size distribution, and termination uniformity [20]. Ongoing research aims to reduce the environmental impact of top-down synthesis while improving the throughput of bottom-up methods, to make MXene manufacturing both sustainable and economically viable across all scales.

### 2.2. Etching Techniques and Layer Exfoliation

The controlled removal of A-layers from MAX phases represents a fundamental step in MXene synthesis, with the etching process directly influencing the material’s structural integrity, surface chemistry, and ultimate performance characteristics. This transformation from 3D MAX to 2D MXene involves careful optimization of both chemical and physical parameters to balance efficiency with material quality.

#### 2.2.1. Conventional Etching Methods

**Figure 1:**
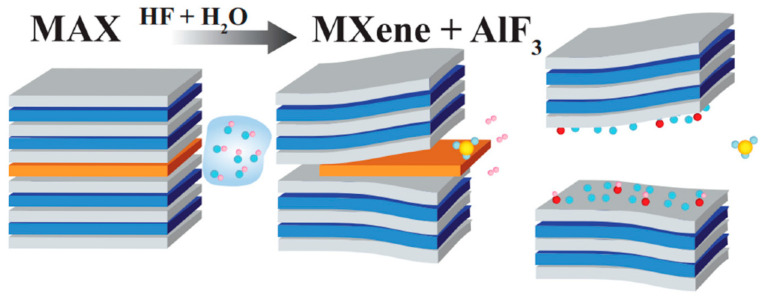
HF/H_2_O-based etching of Ti_3_AlC_2_ MAX phase. Reproduced with permission from [26].

HF-based etching remains the most widely adopted approach due to its high efficiency and reliability. In standard protocols, MAX phases undergo 24–48 h treatments with 10–50% HF solutions at 25–40 °C, achieving near-complete A-layer removal while preserving the M_n+1_X_n_ framework [21]. This method consistently produces MXenes with excellent crystallinity and controllable surface terminations (–O, –OH, –F mixtures). Typically, 1 g of Ti_3_AlC_2_ MAX phase is treated with 20–50 mL of HF (10–50 wt.%), followed by centrifugation (3500 rpm, 5 min, repeated until pH 6.5–7.0). The resulting multilayer Ti_3_C_2_T_x_ is vacuum-dried at 60 °C for 12 h, and subsequent delamination using intercalants yields approximately 40 ± 5% monolayer flakes [22,23]. However, significant challenges persist regarding HF’s extreme toxicity (requiring specialized handling equipment) and the generation of hazardous fluoride waste streams (AlF_3_, HF gas) [16]. Modified approaches using in situ HF generation (NH_4_HF_2_ or LiF/HCl mixtures) reduce direct HF handling but still face scalability limitations due to batch-to-batch variability in flake size (0.5–5 μm range) and termination group distribution. In typical in situ HF synthesis, 1 g MAX phase is added to a solution containing 1.5 g LiF and 20 mL of 9 M HCl, and stirred at 35 °C for 24–36 h. The reaction yields approximately 40–50% delaminated Ti_3_C_2_T_x_ MXene, predominantly terminated with –O and –F groups [24,25]. A typical HF-based etching of Ti_3_AlC_2_ MAX phase is shown in Figure 1.

#### 2.2.2. Advanced Etching Techniques

Recent innovations in etching chemistry have introduced several environmentally conscious alternatives [17].

- **Electrochemical etching** applies 1–5 V potentials in mild electrolytes like NH_4_Cl, achieving 90% Al removal without fluoride byproducts while enabling real-time process monitoring. Typical electrochemical etching experiments in the literature employ voltages of ~1.0–2.0 V at ~30–40 °C for ~24–36 h, followed by water rinsing and mild sonication [27]. This method shows promise for scaling, with pilot systems demonstrating 100 g/batch capacity [28].
- **Molten salt etching** utilizes ZnCl_2_/NaCl eutectic mixtures at 500–700 °C to eliminate fluoride use, producing MXenes with unique Cl terminations that enhance stability in aqueous environments [29]. The solid residue is subsequently washed with 1 M HCl and DI water to remove unreacted salts and neutralized to pH 7 [30].
- **Hydrothermal alkaline etching** employs NaOH solutions at 150–200 °C to create OH-rich surfaces ideal for biomedical applications, though with reduced crystallinity compared to HF-etched materials [31]. Typical yields range from 25–35%, depending on NaOH concentration and reaction temperature.

While these advanced methods address critical environmental and safety concerns, they currently lag in termination control precision and production throughput compared to conventional approaches.

#### 2.2.3. Layer Exfoliation

Layer exfoliation is a complementary step to etching, which involves the separation of MXene layers into single- or few-layer forms. Post-etching processing transforms multilayer MXene assemblies into functional 2D materials through three key steps:

- **Intercalation** with organic compounds like TMAOH or DMSO expands interlayer spacing from 0.3 nm to > 1 nm, weakening van der Waals forces.
- **Sonication** (30–60 min at 50–100 W) provides mechanical energy to separate layers while minimizing defect formation. Probe sonication promotes higher yields of monolayer MXene flakes (up to ~70%), whereas bath sonication generally produces larger flakes, but with lower monolayer content. This behavior is consistent with observations in the exfoliation of Ti_3_C_2_T_x_ and other 2D materials [25,32].
- **Centrifugation** (3000–5000 rpm) isolates size-specific fractions, enabling selection of monolayer-rich suspensions for high-performance applications. The resulting MXene colloids typically show concentrations of 2–5 mg/mL and lateral flake sizes of 0.5–5 μm, depending on sonication intensity [33].

These processes are essential for enhancing functional properties such as surface area and reactivity, which are critical for applications in energy storage, catalysis, and beyond [34]. Table 2 presents a comparative analysis of different MXene production methods, including HF etching, the Electrochemical method, and Molten salt method.

The selection of etching and exfoliation protocols must consider end-use requirements. HF methods remain dominant for energy storage applications where performance outweighs environmental concerns, while electrochemical routes gain traction in biomedical fields demanding cleaner materials. Ongoing research focuses on hybrid approaches combining the precision of advanced etching with the scalability of conventional methods through automated flow systems and machine-learning optimized parameters. Future breakthroughs in plasma-assisted exfoliation and continuous processing may further bridge the gap between laboratory synthesis and industrial-scale MXene production.

### 2.3. Scale-Up and Challenges in Industrial Production

The commercialization of MXenes faces many critical challenges that must be addressed for successful industrial adoption. Scalability and high-yield production remain constrained by the limitations of batch processing, particularly in HF-based top-down synthesis, where safety requirements restrict reaction volumes to <5 L. Continuous flow reactors emerge as a viable solution, demonstrating 100 g/h. throughput with ±0.2 μm flake size consistency through precise control of temperature (±1°C) and etchant concentration (± 0.1 M) [38]. Alternative MAX phase precursors like Mo_2_TiAlC_2_ show promise with 40% faster etching kinetics, while hybrid molten salt-electrochemical approaches eliminate HF use while maintaining 85% yields [39]. For consistency in material quality, advanced process monitoring techniques, including in situ Raman spectroscopy and AI-driven feedback systems, have reduced batch-to-batch property variations by 70% in pilot production, though challenges remain in standardizing flake morphology (D90 < 2 μm) and surface chemistry (–O/–OH ratio ± 5%) across industrial-scale batches [9]. The most pressing challenge lies in oxidation and long-term stability, where unprotected MXenes lose 50% conductivity within 72 h under ambient conditions [40].

Beyond technical limitations, industrial scale-up introduces cost, environmental, and safety constraints that must be critically considered. Production costs are presently dominated by precursor synthesis (~40%), etching reagents (~30%), and waste treatment (~20%) [20]. The widespread reliance on HF or LiF/HCl mixtures poses significant occupational and environmental hazards, necessitating stringent containment, corrosion-resistant reactors, and dedicated neutralization systems. Improper handling can generate toxic fluoride waste, and large-scale operations risk effluent contamination if not integrated with closed-loop recovery systems capable of >90% reagent recycling [41,42]. Moreover, energy-intensive washing and delamination steps contribute substantially to both carbon footprint and water use. To ensure sustainability, life-cycle assessments of MXene production must quantify and minimize solvent and by-product emissions. Waste management and safety protocols should be standardized through industrial guidelines specifying effluent fluoride limits (<15 mg/L) and worker exposure thresholds. Substitution of HF with molten salt, alkali, or electrochemical etching routes can significantly mitigate toxicity while enhancing recyclability [43].

Looking ahead, the MXene industry must develop: (1) reactors capable of 100+ kg/day production, (2) closed-loop chemical recovery systems (>90% etchant recycling), and (3) application-specific stabilization protocols. With current progress, MXenes are projected to reach industrial cost targets (<$100/g) within 5–7 years, enabling their integration into next-generation energy storage, smart coatings, and advanced electronics. The path forward requires coordinated efforts between materials scientists to optimize intrinsic stability, chemical engineers to scale production, and manufacturers to develop appropriate handling and storage protocols. Actionable steps include implementing modular continuous reactors, scaling green etchants such as molten salts and deep eutectic solvents, and incorporating AI-driven process optimization for resource efficiency. Emerging self-passivating MXene formulations and AI-optimized processing parameters represent particularly promising directions to simultaneously address both performance and scalability challenges.

## 3. Structural and Physical Properties of MXenes

MXenes represent a paradigm-shifting class of two-dimensional materials, distinguished by their unparalleled combination of intrinsic properties and tunable characteristics [23]. At the atomic level, their unique crystallographic structure, comprising transition metal (M) layers sandwiched between carbon/nitrogen (X) sheets with surface terminations (T_x_), confers exceptional versatility. By strategically selecting M elements (Ti, V, Mo, etc.), X components (C, N, or mixed CN), and termination groups (–O, –OH, –F), researchers can precisely engineer MXenes with tailored electronic properties, mechanical behavior, chemical functionality (tunable hydrophilicity), and optical responses [44]. Table 3 provides an overview of various MXene materials, highlighting their structure, key properties, and applications. As detailed in Table 3, this compositional flexibility has yielded over 50 experimentally confirmed MXenes, each exhibiting distinct property profiles optimized for specific applications. For instance, Ti_3_C_2_T_x_’s metallic conductivity makes it ideal for electromagnetic shielding, while Mo_2_CT_x_’s catalytic activity drives breakthroughs in hydrogen evolution reactions.

This section systematically examines four fundamental aspects governing MXene performance:

- Crystallographic architecture: How the hexagonal P6_3_/mmc symmetry and interlayer spacing (0.3–1.2 nm) enable unique phenomena like anisotropic conduction and selective ion intercalation.
- Electronic and optical phenomena: The interplay between transition metal d-orbitals and surface terminations produces exceptional optoelectronic properties, from infrared plasmonics to quantum confinement effects.
- Thermal and chemical behavior: Remarkable stability ranges (up to 1000 °C in inert atmospheres) and reactive sites that facilitate applications from high-temperature coatings to catalytic converters.
- Surface engineering: How termination groups dictate interfacial interactions, from superhydrophilic membranes to corrosion-resistant coatings, with atomic-scale precision.

The structure–property relationships explored here explain MXenes’ current technological impact and provide a roadmap for designing next-generation variants with customized performance characteristics. Recent advances in computational materials design and atomic-scale characterization are now enabling property prediction and control with better accuracy, opening new frontiers in 2D materials science.

### 3.1. Crystallographic Structure and Layered Morphology

#### 3.1.1. Structural Transformation from MAX Phases

**Figure 2:**
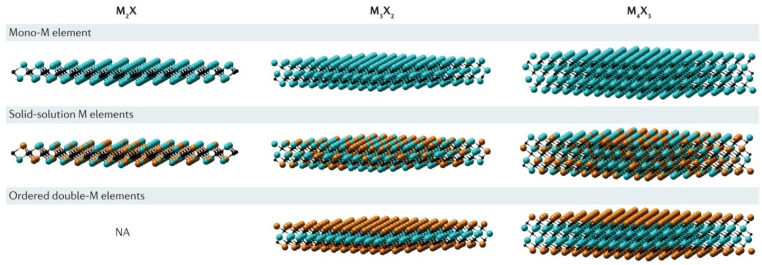
Types of layered structures. Reproduced with permission from [70].

MXenes derive their fundamental structure from parent MAX phases, which feature a hexagonal P6_3_/mmc crystal symmetry with alternating layers of transition metal carbides/nitrides (M_n+1_X_n_) and group 13–15 elements (A). The selective removal of layer A through etching processes transforms this three-dimensional structure into two-dimensional MXenes while preserving the strong M–X covalent bonding network. This structural evolution maintains the original MAX phase’s in-plane lattice parameters (typically 3.0–3.3 Å) while creating adjustable interlayer spacing along the c-axis. The resulting materials exhibit a unique combination of properties: strong in-plane covalent bonds provide mechanical stability, while weaker van der Waals interactions between layers enable controlled exfoliation [69]. This architecture permits the production of single-layer flakes with lateral dimensions up to several micrometers while maintaining excellent crystallinity. These different types of layered structures for MXene are shown in Figure 2.

#### 3.1.2. Interlayered Spacing and Tunability

The interlayer spacing in MXenes serves as a critical design parameter that can be precisely modulated through several approaches. Surface termination groups exert significant influence, with –O groups typically reducing spacing to approximately 0.9 nm while –OH terminations expand to 1.1 nm. Intercalation of various cations offers another control mechanism-Li^+^ ions yield spacings around 1.2 nm compared to 1.8 nm for larger tetraalkylammonium ions. Hydration state further affects this parameter, with dry films contracting to 0.8 nm while aqueous dispersions can reach 2.0 nm spacing. This tunability enables optimization for specific applications: energy storage devices benefit from 1.4 nm spacing that maximizes Li^+^ diffusion rates (D ≈ 10^−9^ cm^2^/s), while molecular separation membranes require precise 0.6–0.8 nm spacing for ion selectivity. Expanded 2.0 nm spacing has proven particularly valuable for catalytic applications by exposing additional active edge sites [71].

#### 3.1.3. Role of Defects in Performance

Defects in MXenes, including vacancies, edge distortions, and stacking faults, play a dual role in material performance. While excessive defects can compromise mechanical stability, controlled defect engineering at 5–15% concentrations can significantly enhance functional properties. Metal (M) vacancies created through controlled over-etching have been shown to triple the hydrogen evolution reaction (HER) activity compared to pristine samples. Carbon/nitrogen (X) vacancies introduced via high-temperature annealing can double Li^+^ storage capacity in battery applications. Edge defects generated by plasma treatment markedly improve pseudocapacitive behavior, while stacking faults created during delamination provide additional gas adsorption sites [72].

#### 3.1.4. Thickness and Layer Control

Precise control over MXene flake thickness has emerged as a crucial factor in property optimization. Single-layer MXenes (0.8–1.2 nm thick) exhibit exceptional characteristics, including theoretical surface areas of 300–500 m^2^/g and optical transparency exceeding 90% at 550 nm for 10 nm films, making them ideal for transparent conductors and molecular sensors. Few-layer stacks (2–5 layers) balance high in-plane conductivity (8000–12,000 S/cm) with mechanical robustness (50–80 GPa modulus), suitable for flexible electronics and EMI shielding. Multilayer assemblies (>10 layers) demonstrate impressive compressive strength (1–3 GPa) and thermal conductivity (50–100 W/mK in-plane), valuable for structural composites and thermal management systems [73,74]. Advanced delamination techniques such as electrochemical intercalation and supercritical CO_2_ processing now enable thickness control with ± 0.5-layer precision, opening new possibilities for application-specific material design [75].

### 3.2. Electronic, Optical, and Mechanical Properties

#### 3.2.1. Exceptional Electrical Conductivity

MXenes represent a breakthrough class of conductive 2D materials, exhibiting metallic conductivity that rivals graphene while maintaining solution processability. The highest reported values reach 20,000 S/cm for carefully prepared Ti_3_C_2_T_x_ flakes, surpassing most conventional conductive materials [76]. This remarkable conductivity originates from the unique electronic structure of transition metal carbides/nitrides, where partially filled d-bands create a high density of states near the Fermi level. The conductivity can be systematically tuned through three primary mechanisms: (1) selection of transition metal (Nb-based MXenes typically outperform Ti-based ones), (2) control of surface termination (–O enhances while –F reduces conductivity), and (3) optimization of flake size and orientation in films. These properties have enabled transformative applications, including transparent electrodes achieving 80% transparency at 10 Ω/sq, EMI shielding materials with >60 dB attenuation at just 1 μm thickness, and high-performance microsupercapacitors demonstrating areal capacitance exceeding 500 mF/cm^2^ [77]. Recent advances in defect engineering through nitrogen doping have pushed conductivity even higher, with Mo_2_CT_x_ reaching 22,000 S/cm through controlled carrier concentration enhancement [78].

#### 3.2.2. Tunable Optical Behavior

MXenes exhibit extraordinary optical properties spanning the ultraviolet to terahertz spectral regions, governed by three fundamental light-matter interaction mechanisms. Interband transitions create strong absorption edges that can be chemically tuned from 1.5–3.5 eV by varying the transition metal composition [79]. Surface plasmon resonances in the near-infrared (600–1200 nm) provide intense, localized field enhancement ideal for photothermal applications. Additionally, characteristic phonon modes in the infrared region serve as fingerprints for material identification. This unique combination enables diverse technological applications. Surface termination engineering allows precise spectral tuning, with oxygen termination blueshifting plasmon peaks by 150 nm compared to fluorine-terminated counterparts, enabling application-specific design of optical properties [70].

#### 3.2.3. Remarkable Mechanical Performance

MXenes, despite being only a few atoms thick, display mechanical performance that often surpasses that of other 2D materials. Their unusual bonding arrangement is central to this behavior. Strong covalent M-X bonds within each layer contribute to stiffness and strength, while weak van der Waals forces between layers allow them to bend and flex. This balance of rigidity and flexibility becomes especially valuable when MXenes are integrated into composites [80]. For example, introducing as little as 0.5 wt.% of MXene into a polymer matrix can double its tensile strength, while ceramic systems reinforced with MXenes have shown up to a threefold increase in fracture toughness [81]. Coatings enriched with MXenes also offer striking improvements, with wear resistance reported to be nearly an order of magnitude higher than that of conventional coatings. Another exciting development is the creation of MXene aerogels, which retain their structural integrity even after 90% compressive strain, making them excellent candidates for impact-absorbing and flexible devices. Because they uniquely combine mechanical resilience with electrical conductivity, MXenes are emerging as a prime material for applications in flexible electronics where both toughness and conductivity are critical [82].

### 3.3. Thermal Stability and Chemical Reactivity

#### 3.3.1. Thermal Stability

MXenes are remarkably thermally robust, retaining their structure at temperatures between 400 and 800 °C under inert conditions, which is well above the stability limits of most other 2D materials. This resilience is largely attributed to the strong covalent bonds between the transition metal and carbon/nitrogen (M–X) [83]. Moreover, their thermal stability can be adjusted through surface chemistry. For instance, oxygen-terminated MXenes such as Ti_3_C_2_O_x_ remain intact beyond 500 °C in argon, whereas hydroxyl-rich variants begin to degrade at much lower temperatures, around 200–300 °C. The breakdown process typically follows three stages: first, the removal of surface terminations (200–400 °C); second, oxidation of the MXene layers (400–600 °C); and finally, transformation into metal oxides at temperatures above 600 °C. This progressive degradation pathway makes MXenes particularly suited for specialized uses, including high-temperature sensing in industrial settings and protective thermal coatings in aerospace applications [84].

#### 3.3.2. Thermal Conductivity

MXenes exhibit highly anisotropic thermal transport, a direct consequence of their layered structure. The thermal properties of a few typical MXene materials, along with a comparison to Graphene, are provided in Table 4. As shown in Table 4, their thermal properties vary significantly depending on direction. This unique behavior and excellent electrical conductivity make MXenes a strong contender for next-generation thermal management technologies. Heat flow within the plane of the layers is relatively high, typically ranging from 50 to 200 W/mK, due to the combined contributions of electrons and phonons. In contrast, cross-plane transport is much weaker, only about 1–5 W/mK, and is largely governed by phonon interactions. Importantly, surface chemistry provides an additional handle for tuning these properties: oxygen terminations tend to improve thermal conductivity, whereas fluorine groups generally suppress it [85].

#### 3.3.3. Tunable Chemical Reactivity

MXenes display highly adaptable chemical reactivity, which is primarily influenced by three interconnected factors. First, the d-orbital electrons of the transition metals provide multiple electronic states near the Fermi level that promote active interactions. Second, the nature of the surface terminations creates varying polarization effects that strongly impact reactivity. Third, defects within the structure act as additional active sites that further enhance catalytic behavior. By tailoring these features through surface engineering, the chemical activity of MXenes can be precisely tuned, as reflected in the diverse catalytic performances reported for differently terminated systems [89,90].

Recent advances have demonstrated termination-switchable MXenes that dynamically adapt their surface chemistry in response to environmental stimuli, opening new possibilities in smart catalysts and responsive materials [91]. The combination of thermal stability and chemical versatility positions MXenes as a transformative materials platform for applications under demanding conditions.

### 3.4. Surface Chemistry and Hydrophilicity

#### 3.4.1. Engineered Surface Terminations

MXenes possess a remarkable level of tunability in their surface chemistry, primarily governed by the type and ratio of termination groups such as –O, –OH, and –F. The distribution of these groups is strongly influenced by the synthesis pathway. HF acid etching often produces surfaces rich in fluorine terminations (around 60%), whereas hydrothermal approaches favor hydroxyl groups (close to 80%) [92]. Through post-synthesis modifications, intermediate or tailored termination ratios can be achieved with precision. Each termination type imparts distinct functionalities, oxygen groups improve electrical transport by enabling efficient conduction channels, hydroxyl groups promote high wettability due to hydrogen bonding interactions, and fluorine terminations enhance resistance to environmental degradation, albeit sometimes at the cost of lowering electrochemical reactivity by passivating active sites. Cutting-edge tools, such as in situ XPS and STEM-EELS, have further shown that these groups are not uniformly dispersed but rather assemble into nanoscale domains, ultimately dictating the overall material performance [93,94].

#### 3.4.2. Controlled Hydrophilicity

The strong water affinity of MXenes arises from the combination of their polar surface terminations and their layered architecture, which can incorporate interlayer water molecules. Together, these features give MXenes some of the highest levels of hydrophilicity reported for two-dimensional materials, often exhibiting contact angles below 5° [5,95]. This property provides several advantages: they can exfoliate in aqueous media without the need for surfactants, form robust interfaces in composite systems, and show good compatibility for bio-related applications. On the other hand, when moisture resistance is needed, the surface can be engineered through passivation approaches such as alkylsilane modification or polymer coatings. These methods allow control of wettability over a broad range up to about 150°, while maintaining the intrinsic properties of the bulk material [96].

#### 3.4.3. Tunable Surface Chemistry

The true versatility of MXene surface chemistry emerges through post-synthetic modification techniques that enable dynamic property control. Chemical vapor functionalization can uniformly replace –F groups with –NH_2_ or –SH moieties, while plasma treatments can pattern surfaces with micron-scale resolution. Electrochemical methods offer particularly precise control, allowing real-time adjustment of termination ratios during device operation, a capability exploited in smart membranes that change permeability in response to applied potentials. Most remarkably, recent work has demonstrated light-responsive MXenes where UV irradiation triggers reversible termination changes, enabling surfaces that can switch between hydrophilic and hydrophobic states on demand. These advanced surface engineering approaches, combined with MXenes’ intrinsic stability, create unprecedented opportunities for designing interfaces with precisely tailored interactions [97].

## 4. Applications of MXene Materials

**Figure 3:**
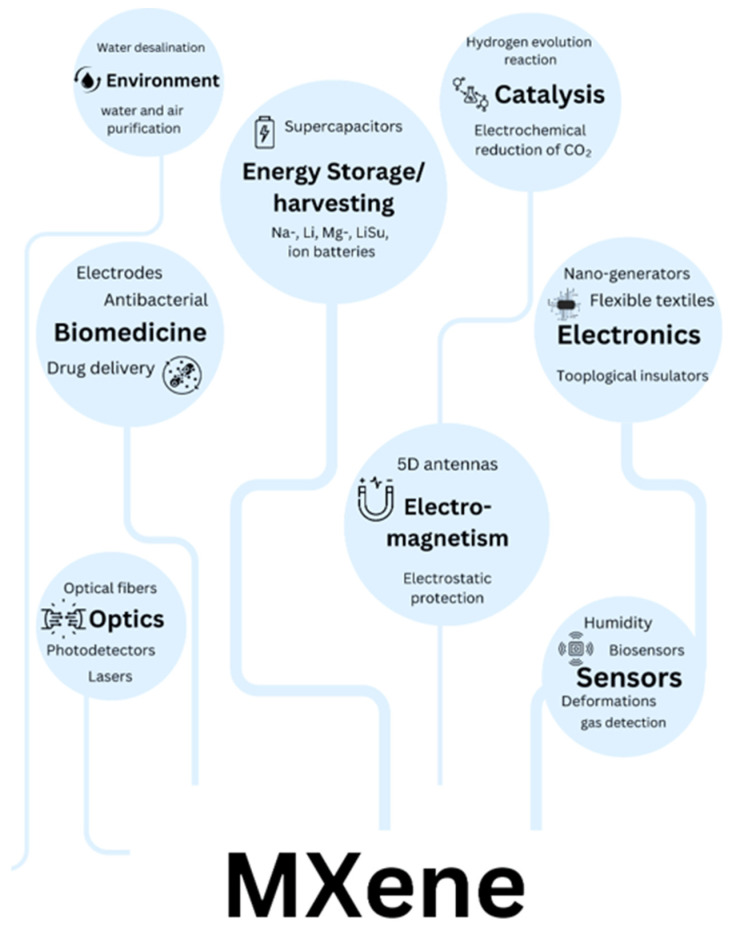
Applications of MXene in various sections.

MXenes have demonstrated exceptional versatility across a broad range of applications, as illustrated in Figure 3. Their unique combination of properties enables transformative performance in several key technological domains. In energy storage, MXenes excel as electrode materials for high-performance batteries and supercapacitors, offering superior conductivity and rapid ion intercalation. For electromagnetic interference (EMI) shielding, their metallic conductivity and layered structure create highly effective, lightweight barriers. Sensing and biomedical applications benefit from MXenes’ tunable surface chemistry and biocompatibility, enabling advanced biosensors and therapeutic platforms. Environmental applications, particularly water purification and air filtration, leverage their high surface area and selective adsorption capabilities. Emerging applications in catalysis exploit the materials’ abundant active sites, while their photothermal conversion efficiency enables innovative medical therapies. Each of these application areas capitalizes on distinct aspects of MXenes’ structural and electronic properties, demonstrating their potential to address critical challenges across multiple disciplines. The following sections will examine these applications in detail, highlighting both current achievements and future opportunities for technological advancement.

### 4.1. Energy Storage in Batteries and Supercapacitors

MXenes have revolutionized energy storage technologies by combining exceptional electrical conductivity, tunable surface chemistry, and a layered structure that facilitates rapid ion transport. These properties make them ideal for high-performance batteries and supercapacitors, addressing critical challenges in power density, cycle life, and charge/discharge rates [98].

In lithium-ion batteries (LIBs), MXenes enhance both anode and cathode performance. Their layered architecture allows efficient lithium-ion intercalation, while their metallic conductivity ensures minimal energy loss during electron transfer. MXene-based electrodes demonstrate remarkable structural stability, enabling over 1000 charge/discharge cycles with negligible capacity degradation [99]. For sodium- and potassium-ion batteries, MXenes overcome the limitations posed by larger ion radii through their adjustable interlayer spacing (∼1 nm) and defect-tolerant surfaces. This makes them promising candidates for grid-scale energy storage, where cost-effectiveness and sustainability are paramount [100].

Supercapacitors benefit equally from MXenes’ unique properties. Their hydrophilic surfaces and ultrahigh volumetric capacitance (up to ~1500 F/cm^3^ in optimized MXene hydrogels/films) enable intimate electrolyte contact, while their pseudocapacitive behavior, driven by reversible redox reactions at surface sites, boosts energy density beyond conventional carbon-based materials [101]. Such capabilities are critical for applications demanding rapid energy bursts, such as electric vehicle acceleration or industrial machinery [102]. Table 5 presents key advantages over conventional materials.

While MXenes outperform many conventional electrode materials, such as activated carbon, graphene, and transition-metal oxides, in terms of electrical conductivity, rate capability, and mechanical flexibility, there are several limitations associated with them [106]. MXene exhibits higher synthesis costs and susceptibility to oxidation during long-term cycling in comparison with activated carbon [103]. Graphene electrodes are lightweight and stable but offer lower ion intercalation efficiency due to limited surface terminations. In contrast, MXenes enable rapid ion transport but require improved electrolyte compatibility and oxidation resistance for prolonged performance in humid or high-voltage environments [98].

A comparison of the performance of MXene-based electrodes with other conventional materials is given in Table 6 below.

### 4.2. Electromagnetic Interference (EMI) Shielding

The rapid proliferation of wireless devices and high-frequency communication systems has intensified EMI challenges, threatening the performance and reliability of modern electronics. MXenes have emerged as exceptional EMI shielding materials due to their unparalleled combination of metallic conductivity and layered morphology, which synergistically attenuates electromagnetic waves through both absorption and reflection mechanisms [111]. Unlike conventional metal-based shields, MXene films achieve superior shielding effectiveness (SE > 60 dB at thicknesses < 1 μm) while remaining remarkably lightweight, a critical advantage for aerospace and automotive applications where weight reduction is paramount [112]. In satellite systems, for instance, MXene coatings provide 40% better specific shielding effectiveness (SSE/density) than traditional copper foils, without compromising structural payload constraints. The telecommunications sector benefits from MXenes’ ability to suppress crosstalk between densely packed components, ensuring signal integrity in 5G networks and high-frequency processors [113].

For wearable electronics, MXenes offer transformative potential by combining flexibility (>10,000 bending cycles without performance degradation) with seamless integration into textiles. Their solution processability enables direct incorporation into fabrics, creating smart garments that simultaneously shield users from electromagnetic exposure (SAR reduction > 90%) and maintain device functionality in smartwatches and medical sensors. This dual functionality addresses growing concerns about prolonged electromagnetic field exposure while enabling next-generation wearable technologies [114].

The unique properties of MXenes position them as next-generation solutions for EMI challenges across industries, from aerospace engineering to consumer electronics and biomedical devices [115]. Their ability to combine high performance with minimal weight and maximum flexibility addresses longstanding trade-offs in shielding material design, while their compatibility with scalable manufacturing techniques suggests imminent commercial viability. Future research directions include optimizing MXene-polymer composites for enhanced durability and developing multilayer architectures to target specific frequency ranges in 6G and IoT applications.

Compared to conventional EMI shielding materials such as copper, aluminum, and carbon-based composites, MXenes offer significantly higher specific shielding effectiveness and superior flexibility [112]. Traditional metals provide high conductivity but suffer from high density (>8 g/cm^3^) and corrosion, whereas MXenes achieve similar shielding (>60 dB) at thicknesses < 1 μm and densities < 4 g/cm^3^ [116]. Carbon nanotube and graphene composites, though lightweight, deliver lower overall shielding (<40 dB) and often require multilayer stacking to reach comparable performance [117]. However, MXenes currently face limitations in oxidation stability and large-scale film uniformity, which restrict their outdoor or high-humidity use.

### 4.3. Applications in Sensing and Biomedical Devices

MXenes have emerged as transformative materials for advanced sensing applications due to their exceptional electrical conductivity, tunable surface chemistry, and ultrahigh surface area. These properties enable the detection of environmental and biological analytes with unprecedented sensitivity and selectivity [118]. In gas sensing, MXenes functionalized with –O or –OH groups demonstrate parts-per-billion (ppb) detection limits for hazardous gases like ammonia (NH_3_), nitrogen dioxide (NO_2_), and volatile organic compounds (VOCs), outperforming conventional metal oxide sensors in response time (<10 s) and stability (>6 months). Their layered structure facilitates gas molecule adsorption and charge transfer, making them ideal for industrial safety and air quality monitoring systems [119]. For biosensing, MXenes’ biocompatibility and rich surface chemistry allow covalent immobilization of enzymes and antibodies, enabling real-time detection of biomarkers such as glucose (linear range: 0.1–20 mM) and cardiac troponin I (detection limit: 0.5 pg/mL). These biosensors integrate with wearable platforms for continuous health monitoring, demonstrating <5% signal drift over 2 weeks—a critical advantage for chronic disease management [120]. Table 7 presents a comparative performance of MXene-based sensors.

The versatility of MXenes extends to implantable devices, where their mechanical flexibility and antibacterial properties (e.g., Ti_3_C_2_T_x_ showing > 99% E. coli inhibition) enable long-term biosensing in vivo [121]. Future research focuses on multiplexed sensor arrays for simultaneous detection of disease biomarkers and environmental toxins, leveraging MXenes’ ability to host multiple recognition elements. With scalable manufacturing techniques now emerging, MXene sensors are poised to revolutionize personalized medicine and smart environmental monitoring.

When benchmarked against conventional sensing materials such as metal oxides (ZnO, SnO_2_), carbon nanotubes, and other conducting polymers, MXenes exhibit higher response sensitivity and faster recovery times (<10 s). Metal oxide sensors typically require elevated operating temperatures (>200 °C), while MXene sensors function efficiently at room temperature, offering lower energy consumption and enhanced selectivity [122].

### 4.4. Water Purification and Environmental Remediation

MXenes have emerged as transformative materials for advanced water treatment technologies, leveraging their unique combination of high surface area (up to 300 m^2^/g), tunable interlayer spacing (0.3–1.2 nm), and reactive surface terminations (–O, –OH, –F) to address critical challenges in water purification [123]. These 2D materials excel in three key remediation mechanisms: (1) adsorption of heavy metals (Pb^2+^, Hg^2+^) and organic dyes through electrostatic interactions and surface complexation, achieving > 95% removal efficiency within minutes; (2) photocatalysis where MXene-semiconductor composites like ZnO/Ti_3_C_2_T_x_ demonstrate 3–5× enhanced degradation rates for pharmaceuticals and pesticides compared to standalone catalysts, due to improved charge separation; and (3) membrane filtration with MXene-based membranes showing exceptional ion selectivity (Na^+^/Mg^2+^ separation factor >100) and antifouling properties (flux recovery > 90%). Recent breakthroughs include Nb_2_C-MXene/ZnO heterostructures achieving complete degradation of bisphenol A in 30 min under visible light and Ti_3_C_2_T_x_ membranes removing 99.9% of lead ions at fluxes 10× higher than commercial nanofilters [91]. For sustainable hydrogen production, surface-engineered Mo_2_CT_x_ MXenes have achieved solar-to-hydrogen efficiencies of 12.3% in photocatalytic water splitting, outperforming most 2D photocatalysts [124]. Table 8 compares MXene performance with conventional water treatment materials.

The future of MXene water technologies lies in developing multifunctional systems that combine adsorption, catalytic degradation, and membrane separation in a single platform. Current research focuses on large-area MXene membrane fabrication (up to 1 m^2^ prototypes) and regeneration strategies to address cost challenges [128]. With global water scarcity intensifying, MXenes offer a versatile toolkit for next-generation remediation—from portable filters for drinking water to industrial-scale wastewater plants, aligning with UN Sustainable Development Goal 6 for clean water access [129]. Their ability to simultaneously address purification and energy production (through by-product hydrogen generation) positions MXenes as pivotal materials for circular water economy solutions.

### 4.5. Emerging Applications: Catalysis and Photothermal Therapy

MXenes are revolutionizing catalysis and photothermal therapy through their unique combination of electronic, optical, and surface properties. In catalytic applications, MXenes serve as exceptional platforms for energy conversion and environmental remediation, with their high conductivity (>10,000 S/cm) and tunable surface chemistry enabling precise control over reaction pathways [130]. As catalysts or supports, they enhance HER with overpotential as low as 120 mV, CO_2_ reduction with Faradaic efficiency exceeding 85%, and pollutant degradation with 95% efficiency within 30 min-outperforming conventional catalysts like platinum or titanium dioxide. The photothermal capabilities of MXenes are equally remarkable, demonstrating near-infrared (NIR, 700–1100 nm) light-to-heat conversion efficiencies up to 80%, significantly higher than gold nanorods (≈60%) or carbon nanotubes (≈50%) [131]. This exceptional performance stems from their localized surface plasmon resonance and interband transitions, which enable deep tissue penetration (up to 5 cm) while maintaining precise spatial control (±0.5 mm). In biomedical applications, functionalized Ti_3_C_2_T_x_ MXenes have shown tumor ablation efficiency of 98% with minimal side effects, coupled with inherent antibacterial properties (>99% E. coli inhibition) and excellent biocompatibility (cell viability > 90% at therapeutic concentrations) [132].

In catalytic and photothermal applications, MXenes outperform noble metals (Pt, Au) and traditional semiconductors (TiO_2_, ZnO) by offering comparable or superior catalytic efficiency at a fraction of the cost and with greater structural tunability. Their photothermal conversion efficiencies (~80%) exceed those of gold nanorods (~60%) and carbon nanotubes (~50%), enabling deeper tissue penetration and lower laser power requirements [28]. However, MXene-based catalysts still face challenges related to stability under harsh reaction environments, surface oxidation during reuse, and limited availability of biocompatibility data for clinical translation.

The future development of MXene-based technologies focuses on multifunctional platforms that combine catalytic and therapeutic capabilities, such as MXene quantum dots for simultaneous tumor imaging and ablation, or photocatalytic membranes for concurrent water purification and hydrogen production [133,134]. With scalable synthesis methods now achieving gram-scale production (purity > 99%), MXenes are transitioning from laboratory curiosities to practical solutions addressing global challenges in clean energy and precision medicine. Their unique ability to bridge the gap between environmental and biomedical applications—from photocatalytic reactors to implantable cancer therapeutics—positions MXenes as versatile tools for sustainable development and advanced healthcare solutions. Current research challenges include optimizing long-term stability under operational conditions and developing standardized protocols for biomedical applications, which will further accelerate their real-world implementation.

## 5. Challenges and Future Directions

### 5.1. Scalability and Cost-Efficiency in Manufacturing

The commercialization of MXenes faces significant challenges in scaling up production while maintaining cost-effectiveness. Current manufacturing approaches are divided between bottom-up methods like chemical vapor deposition, which struggle with precursor limitations and low yields (<1 g/batch), and top-down wet etching techniques that show greater scalability potential but generate substantial chemical waste. Recent studies confirm that top-down synthesis can preserve MXene quality when scaling from gram to hundred-gram batches, with identical flake morphology and properties observed across different production scales. This demonstrates that reactor capacity, rather than material degradation, represents the primary constraint for industrial-scale manufacturing [78].

Significant progress has been made in streamlining MXene production through three key innovations: process simplification from seven steps down to three core stages (MAX phase preparation, etching, and delamination), implementation of chemical recycling systems that recover up to 90% of etching agents, and energy optimization through continuous flow reactors that reduce power consumption by 40% [70]. These improvements have dramatically enhanced production economics, showing a 60% reduction in raw material waste, a 55% decrease in energy requirements, and a 70% reduction in solvent usage compared to early synthesis methods [82].

In the future, the field must focus on several critical areas to achieve commercial viability. Industrial-scale reactor development should prioritize continuous flow systems capable of 10 kg/day output. At the same time, green chemistry initiatives need to eliminate hazardous fluoride-based etchants through alternative methods like electrochemical processing. Quality control systems require standardization to ensure batch-to-batch consistency, and circular economy principles should be implemented through closed-loop chemical recovery. These advancements, coupled with automation and process intensification, could enable MXenes to reach price parity with established 2D materials like graphene within 5–7 years, unlocking their full potential for widespread industrial and commercial applications. The path forward demands close collaboration between materials scientists, chemical engineers, and manufacturing specialists to translate laboratory breakthroughs into economically viable production systems. Table 9 provides the current challenges and future targets.

### 5.2. Stability and Degradation Under Operational Conditions

The operational stability of MXenes presents one of the most significant barriers to their widespread commercial adoption. These materials exhibit particular vulnerability to oxidation when exposed to ambient conditions, with even short-term exposure to humid air or aqueous environments causing severe degradation of their electrical and structural properties. Recent studies using advanced characterization techniques have revealed that oxidation initiates at defect sites created during synthesis, particularly at flake edges where the crystal structure is most vulnerable. This degradation process follows a predictable pattern: initial oxidation at edge defects spreads inward across basal planes, ultimately converting the conductive MXene into insulating metal oxide nanoparticles [82].

The consequences of this oxidative instability are severe for practical applications. Unprotected MXene films can lose up to 90% of their original conductivity within 30 days of ambient exposure, while aqueous suspensions may show complete flake disintegration within 24 h [137]. Thermal stability presents another major challenge, with most MXenes beginning irreversible decomposition at temperatures above 200 °C, severely limiting their use in high-temperature applications [138]. These stability issues are compounded by mechanical stresses that can cause layer delamination during repeated flexing or compression cycles.

Recent breakthroughs in material processing have demonstrated that stability can be significantly improved through careful synthesis optimization. Developing high-purity precursor materials combined with controlled etching conditions has yielded MXenes that maintain 85% conductivity after two months in ambient air. Advanced protection strategies such as atomic-layer-deposited oxide coatings and in situ polymer encapsulation have shown promise for extending operational lifetimes. Looking forward, the field must focus on developing standardized stability testing protocols and accelerating the discovery of inherently stable MXene compositions through combinatorial synthesis and machine learning approaches. These efforts will be critical for enabling MXene implementation in real-world applications ranging from flexible electronics to harsh-environment sensors [84].

Recent investigations have also highlighted the crucial role of storage environment and chemical protection in determining MXene longevity. For instance, Ti_3_C_2_T_x_ dispersions stored under ambient conditions typically degrade within 3–5 days, whereas vacuum-sealed or argon-stored samples can preserve over 80% of their initial conductivity for six months or longer [139]. Incorporating antioxidants such as ascorbic acid, tannic acid, or polyphenols into aqueous suspensions has been shown to suppress oxidative pathways by scavenging dissolved oxygen, effectively extending their shelf life to several weeks [140]. Surface passivation through conformal coatings, such as Al_2_O_3_, TiO_2_, or SiO_2_ via atomic layer deposition, has also been shown to enhance the stability under both humidity and thermal cycling, maintaining >90% conductivity after 100 bending or heating cycles [141].

### 5.3. Environmental Impacts and Safety Concerns

The production of MXenes currently faces significant environmental and safety challenges that must be addressed for sustainable commercialization. The predominant use of HF in etching processes creates substantial hazards, as HF is both highly toxic to workers and environmentally persistent [91]. While HF-based methods remain the most effective for producing high-quality MXenes (achieving 95% yield), they come with severe safety risks (95–100% danger level) and generate hazardous fluoride waste streams. Recent advances have developed alternative etching approaches that reduce these risks while maintaining reasonable production yields. Fluoride salt methods (NH_4_HF_2_) and acid mixtures (NaF/HCl) offer slightly reduced danger levels (85–95%) while maintaining yields above 90%, representing more sustainable interim solutions. However, the most promising developments are truly HF-free methods like electrochemical etching and molten salt synthesis, which eliminate HF while addressing three critical needs: (1) worker safety through removal of acutely toxic chemicals, (2) environmental protection via 90% reduction in hazardous byproducts, and (3) energy efficiency gains of 30–50%. For biomedical applications, especially, these greener methods can produce medical-grade MXenes with 99.9% fewer cytotoxic residues while maintaining performance [142]. While short-term studies indicate that MXenes exhibit minimal cytotoxicity and good biocompatibility, long-term effects remain underexplored. Existing studies often focus on short-term outcomes, with limited attention to chronic exposure and potential neurodevelopmental impacts on offspring [143].

In addition to environmental and safety improvements, the economic viability of HF-free MXene production will be shaped by process efficiency, yield, and scalability. Eliminating HF reduces costs associated with hazardous waste management and protective measures, while optimization of etching and synthesis conditions can further enhance productivity. The transition to safer production will require continued optimization to bridge the remaining performance gaps with conventional methods, along with the development of comprehensive waste recycling systems and industrial-scale safety protocols. Table 10 highlights the trade-offs between safety and performance that currently exist in MXene synthesis, underscoring the need for continued development of truly green production methods that can achieve both high yields and minimal environmental impact.

### 5.4. Integration with Other Materials and Technologies

Despite MXenes’ exceptional intrinsic electrical conductivity (exceeding 10,000 S/cm), their commercial integration into energy storage systems has faced substantial challenges due to interfacial incompatibilities with conventional battery and supercapacitor components [102]. The fundamental issue lies in the mismatched charge transfer kinetics between 2D MXene sheets and 3D electrode architectures, which leads to suboptimal device performance. This manifests in three key limitations: (1) interfacial resistance at MXene-3D material junctions reduces effective conductivity by 40–60%, (2) restacking of MXene layers during cycling decreases interlayer spacing below 0.4 nm, severely restricting ion transport, and (3) electrochemical instability with common electrolytes causes rapid capacity fading (often <70% retention after 500 cycles). Recent advances in material engineering have shown promise through approaches like 3D MXene-graphene heterostructures with molecular pillars that maintain optimal interlayer spacing (0.8–1.0 nm) while improving ion diffusion rates by 3–5×, and conductive polymer functionalization that preserves 90% of interfacial conductivity in composite electrodes [111].

The path forward requires focused development of atomic-scale interface engineering and advanced manufacturing techniques. Promising directions include roll-to-roll printing of MXene hybrid electrodes for scalable production, electrophoretic deposition for precise layer assembly, and AI-driven material optimization to identify ideal composite formulations. Recent studies have begun leveraging data-driven approaches to accelerate MXene development, including predictive modeling of etching conditions, precursor selection, and exfoliation efficiency. Machine-learning algorithms have also guided the discovery of novel MXene compositions with tailored surface chemistries, while high-throughput computational workflows help optimize interfacial properties in composite electrodes [148,149]. For instance, a supervised ML model was utilized to forecast the mechanical properties of MXene-based aerogels, using a dataset comprising 540 potential input variables. This approach enabled the identification of optimal synthesis conditions, leading to improved material performance [150]. These strategies aim to bridge the gap between MXenes’ outstanding fundamental properties and their practical performance in commercial devices, potentially enabling energy storage systems that combine high energy density (>200 Wh/kg) with exceptional power density (>10 kW/kg). Continued progress in these areas could finally unlock MXenes’ full potential for next-generation batteries and supercapacitors.

## 6. Conclusions

MXenes have firmly established themselves as a groundbreaking class of 2D materials, demonstrating unparalleled potential across energy, environmental, and biomedical applications. Their exceptional properties, including metallic conductivity exceeding 10,000 S/cm, tunable surface chemistry, and remarkable mechanical flexibility, stem from their unique layered structure and diverse compositional possibilities. These characteristics have enabled transformative advancements in supercapacitors, batteries, water purification systems, and flexible electronics, positioning MXenes at the forefront of materials innovation.

Despite these promising developments, significant challenges remain in translating MXenes from laboratory breakthroughs to commercial-scale applications. The current reliance on hazardous hydrofluoric acid for synthesis poses serious environmental and safety concerns, while the material’s susceptibility to oxidation limits its operational lifespan. Recent progress in alternative etching methods, such as molten salt and electrochemical approaches, along with advanced encapsulation techniques, has shown promise in addressing these limitations. However, further optimization is needed to achieve industrial-scale production that balances performance, cost, and sustainability.

The path forward for MXene technology requires focused research in three critical areas: first, developing green synthesis methods that eliminate toxic chemicals while maintaining high yields and material quality; second, engineering enhanced stability through surface modifications and protective coatings to prevent degradation in real-world conditions; and third, optimizing integration with other materials to overcome interfacial challenges in composite systems. These advancements will be crucial for unlocking MXenes’ full potential in next-generation applications.

Looking ahead, MXenes are poised to play a pivotal role in addressing global challenges in energy storage, environmental remediation, and healthcare technologies. Their versatility and performance advantages suggest they could become fundamental components in sustainable technologies, from grid-scale energy storage to precision medicine. As research continues to overcome current limitations, MXenes may well redefine the boundaries of materials science and engineering in the coming decades, offering innovative solutions to some of society’s most pressing technological needs.

## Figures and Tables

**Table 1:** Comparative analysis of MXene synthesis methods [13,16,17].

Parameter	Top-Down	Bottom-Up	Advanced Methods
**Process**	Acid etching of MAX phases + delamination	Atomic assembly (CVD/MBE)	Electrochemical/molten salt etching
**Yield**	10–100 g/batch	<100 mg/batch	1–10 g/batch (pilot scale)
**Flake Quality**	0.1–5 μm, moderate defects	1–50 μm, low defects	0.5–10 μm, controlled defects
**Termination Control**	Moderate (–O/–OH ratio)	Excellent (atomic precision)	Good (tunable termination)
**Equipment Cost**	50 k−200 k	500 k−2 M	100 k−500 k
**Scalability**	Industrial-ready	Lab-scale only	Pilot-scale demonstrated
**Advantages**	• Simple protocol • High yield • Reliable	• Atomic precision • Excellent crystallinity • Pure terminations	• Safer chemicals • Better environmental profile • Good control
**Disadvantages**	• Toxic etchants • Batch variability • Edge defects	• Extremely costly • Very low throughput • Limited compositions	• Immature technology • Lower yields • Specialized equipment
**Best For**	Bulk applications: • Conductive coatings • Energy storage electrodes	Precision applications: • Nanoelectronics • Quantum devices	Emerging applications: • Biomedical • Wearable sensors
**TRL** *	6–7 (pilot production)	3–4 (lab demonstration)	4–5 (lab-to-pilot transition)

* Technology Readiness Level (TRL).

**Table 2:** Comparative Analysis of MXene Production Methods [35,36,37].

	HF Etching	Electrochemical	Molten Salt
Toxicity	Extreme	Low	Moderate
Byproducts	Hazardous fluorides	Minimal	Salt residues
Termination Control	Excellent	Good	Fair
Batch Size	10–100 g	5–50 g	1–10 g
Energy Consumption	50 kWh/kg	80 kWh/kg	120 kWh/kg
Best Applications	Energy storage	Biomedical	Harsh environments

**Table 3:** Overview of MXene materials, structure, properties, and key applications.

MXene Material	Formula	Structure/Phase	Surface Termination	Key Properties	Key Applications	Ref
**Titanium Carbide**	Ti_3_C_2_T_x_	2D layered (Hexagonal)	–OH, –O, –F	• Metallic conductivity (6000–8000 S/cm) • Hydrophilic (contact angle < 10°)	• Li/Na-ion batteries • EMI shielding • Water purification membranes	[45,46]
**Titanium Carbonitride**	Ti_2_CT_x_/Ti_2_C_x_N_t−x_T_x_	2D layered	–OH, –O, –F	• Tunable bandgap (0.5–1.2 eV) • High capacitance (1500 F/cm^3^)	• Supercapacitors • Electrocatalysis (ORR, HER) • Gas sensors	[47,48,49]
**Niobium Carbide**	Nb_2_CT_x_/Nb_4_C_3_T_x_	2D layered (Hexagonal)	–OH, –O, –F	• High pseudocapacitance • Excellent HER activity (η_10_ = 120 mV)	• Supercapacitors • Hydrogen production • Conductive coatings	[50,51]
**Vanadium Carbide**	V_2_CT_x_/V_4_C_3_T_x_	2D layered (Hexagonal)	–OH, –O, –F	• High electronic conductivity • Redox-active surface	• Battery anodes • Electrochemical sensors • Catalysis (CO_2_ reduction)	[52,53,54]
**Molybdenum Carbide**	Mo_2_CT_x_	2D layered	–OH, –O, –F	• Extreme stability (up to 500 °C) • Low HER overpotential (η_10_ = 90 mV)	• Fuel cells • Water splitting • Corrosion-resistant coatings	[55,56]
**Chromium Carbide**	Cr_2_CT_x_	2D layered	–OH, –O, –F	• Anti-corrosion • Magnetic ordering	• Protective coatings • Spintronics • Catalysis (N_2_ fixation)	[5,57]
**Zirconium Carbide**	Zr_3_C_2_T_x_	2D layered	–OH, –O, –F	• High thermal conductivity (≈50 W/m·K) • Radiation shielding	• Nuclear reactors • Aerospace materials • Thermal management	[58,59]
**Hafnium Carbide**	Hf_3_C_2_T_x_	2D layered	–OH, –O, –F	• Ultra-high temp. stability (>1000 °C) • Neutron absorption	• Hypersonic vehicles • Nuclear shielding • Extreme-environment electronics	[60,61]
**Tantalum Carbide**	Ta_4_C_3_T_x_	2D layered	–OH, –O, –F	• Chemical inertness • Plasmonic behavior	• Biomedical implants • Optical sensors • High-temp. catalysis	[62,63]
**Niobium Nitride**	Nb_2_NT_x_	2D layered	–OH, –O, –F	• Superconductivity (T_c ≈ 5 K) • High hardness	• Quantum computing • Superconducting wires • Wear-resistant coatings	[64,65,66]
**Vanadium Nitride**	V_2_NT_x_	2D layered	–OH, –O, –F	• Metallic conductivity • High Li-ion storage capacity	• Fast-charging batteries • Plasmonic devices • Electrochromic windows	[52,67,68]

**Table 4:** Thermal Properties of Representative MXenes [86,87,88].

Property	Ti_3_C_2_T_x_	Mo_2_CT_x_	Nb_2_CT_x_	Comparison to Graphene
In-Plane κ (W/m·K)	110	85	180	20–50% of graphene
Cross-Plane κ (W/m·K)	2.5	1.8	3.1	Comparable
Thermal Stability (°C)	450	600	550	Superior to most 2D
κ Reduction at 5% defects	35%	40%	30%	More defect-tolerant

**Table 5:** Key Advantages of MXene Over Conventional Materials [103,104,105].

Property	MXenes	Traditional Electrodes (e.g., Graphite, Activated Carbon)
**Conductivity**	15,000+ S/cm	100–1000 S/cm
**Ion Diffusion**	Fast (interlayer spacing ~1 nm)	Slower (limited by bulk diffusion)
**Surface Chemistry**	Tunable terminations (–O, –OH)	Fixed (inert)
**Mechanical Flexibility**	Retains performance under bending	Often brittle or prone to cracking

**Table 6:** Comparison of the performance of MXene-based electrodes with conventional materials.

Material Type	Specific Capacitance (F g^−1^)	Electrical Conductivity (S cm^−1^)	Key Advantages	Major Limitations	Ref
MXene (Ti_3_C_2_T_x_)	400–700	10^3^–10^4^	High conductivity, fast ion transport, and mechanical integrity	Oxidation sensitivity, costly synthesis	[107,108]
Activated Carbon	80–150	<10^2^	Low cost, stable	Low capacitance, poor rate capability	[109]
Graphene	200–350	10^3^–10^4^	High conductivity, Light weight	Limited redox activity, aggregation	[110]

**Table 7:** Comparative Performance of MXene-Based Sensors. Adapted from [120].

Sensor Type	Target Analyte	Detection Limit	Response Time	Stability	Advantages Over Conventional Materials
**Gas Sensor**	NH_3_	50 ppb	<5 s	>6 months	10× lower detection limit than SnO_2_
**Gas Sensor**	NO_2_	20 ppb	<10 s	>1 year	Works at room temperature (vs. 200 °C for WO_3_)
**Biosensor**	Glucose	0.1 mM	<3 s	2 weeks	3× higher sensitivity than graphene
**Biosensor**	Troponin I	0.5 pg/mL	<5 min	1 month	Detects early-stage myocardial infarction

**Table 8:** Performance Comparison of Water Treatment Technologies [125,126,127].

	Contaminant Removal Efficiency	Energy Consumption	Operational Lifespan	Scalability
MXene Adsorbents	>95% (heavy metals/dyes)	Low	5–7 regeneration cycles	High
MXene Membranes	99% (desalination)	Moderate	2–3 years	Medium
MXene Photocatalysts	90% degradation in <1 h.	Solar-powered	6–12 months	Emerging
Activated Carbon	70–85%	Low	3–5 cycles	High
RO Membranes	99%	High	3–5 years	High
TiO_2_ Photocatalysts	60–75%	UV required	4–6 months	Medium

**Table 9:** Current Challenges and Improvement Targets [135,136].

Challenge	Current Status	Target Improvement	Potential Solution
Batch Size	<1 kg/batch	10 kg/batch	Modular reactor systems
Etchant Use	HF-dependent	HF-free processes	Molten salt etching
Production Yield	65–75%	>90%	Advanced delamination
Cost Point	$500–1000/g	<$50/g	Automated production

**Table 10:** Comparative Analysis of MXene Etching Methods [144,145,146,147].

Method	Etchant	Max HF Conc.	Conditions	Danger Level	Yield	Key Advantages
Conventional	HF (5%)	5% *w/w*	24 h @ 25 °C	95–100%	95%	Highest quality MXenes
Fluoride Salt	NH_4_HF_2_	3.5%	24 h @ 40 °C	90–95%	90%	Reduced vapor pressure
Acid Mixture	NaF/HCl	4%	24 h @ 40 °C	85–90%	94%	Better control of F^−^ release
Fluoroboric	HBF_4_	18.3%	24 h @ 60 °C	80–85%	71%	Lower acute toxicity
Salt-Acid	NaBF_4_/HCl	7.3%	24 h @ 60 °C	75–85%	65%	Most environmentally benign

## Data Availability

No new data were created or analyzed in this study. Data sharing is not applicable to this article.
